# Citizens' preferences for the conservation of agricultural genetic resources

**DOI:** 10.3389/fgene.2014.00440

**Published:** 2014-12-18

**Authors:** Eija Pouta, Annika Tienhaara, Heini Ahtiainen

**Affiliations:** MTT Agrifood Research FinlandHelsinki, Finland

**Keywords:** native breeds, native varieties, genetic resources, choice experiment, preference heterogeneity, valuation

## Abstract

Evaluation of conservation policies for agricultural genetic resources (AgGR) requires information on the use and non-use values of plant varieties and animal breeds, as well as on the preferences for *in situ* and *ex situ* conservation. We conducted a choice experiment to estimate citizens' willingness to pay (WTP) for AgGR conservation programmes in Finland, and used a latent class model to identify heterogeneity in preferences among respondent groups. The findings indicate that citizens have a high interest in the conservation of native breeds and varieties, but also reveal the presence of preference heterogeneity. Five respondent groups could be identified based on latent class modeling: one implying lexicographic preferences, two with reasoned choices, one indicating uncertain support and one with a preference for the current status of conservation. The results emphasize the importance of *in situ* conservation of native cattle breeds and plant varieties in developing conservation policies.

## Introduction

The intensification of agriculture has led to marked changes in the utilization of agricultural genetic resources (AgGR), and many previously common cultivated plant varieties as well as native animal breeds that are of interest in terms of food and agricultural production have become rare or even endangered (Drucker et al., 2001; FAO, 2007, 2010). In Finland, several native breeds, such as the Eastern and Northern Finncattle, the Kainuu Gray Sheep and the Åland Sheep, are endangered according to the FAO classification (FAO, 2007), and the majority of old Finnish crop varieties as well as the Finnish landrace pig are already extinct.

Decisions on the focus and extent of genetic resource conservation should consider both the costs and benefits of conservation. The full benefits of conserving AgGR are not revealed by markets, as the resources are either not traded in the markets or the price of agricultural products does not completely capture their value (Oldfield, 1989; Brown, 1990; Drucker et al., 2001). These market failures result in an inefficient allocation of resources, i.e., the level of conservation is too low as the full benefits are not considered. Although the importance of economic analyses has been recognized, the literature on the monetary value of genetic resources in agriculture is still relatively limited (e.g., Evenson et al., 1998; Rege and Gibson, 2003; Ahtiainen and Pouta, 2011).

Conservation policies for AgGR in Finland, as in many other European countries, are currently based on international agreements such as the Convention on Biological Diversity (1992) and the Global Plan of Action for Animal Genetic Resources (FAO, 2007). National genetic resource programmes were initiated for plants in the year 2003 and for farm animals in 2005 to strengthen the conservation of genetic resources in Finland. Although there has been some progress in putting the programmes into action, they have not been fully implemented. This may reflect, for example, the lack of political interest in the conservation.

To evaluate conservation policies, there is a need for monetary benefit estimates that encompass both use and non-use values associated with genetic resources. Use values refer to the benefits obtained from current and future use of genetic resources in production and breeding, while non-use values are generated from the knowledge that genetic resources, e.g., certain breeds, exist and are saved for future generations. Stated preference methods, such as the discrete choice experiment (CE) method, are capable of estimating both use and non-use values in monetary terms. A choice experiment is a survey-based method whereby respondents are asked to choose between two or more discrete alternatives that are described with attributes. By varying attribute levels and including a price variable as one of the attributes, respondents' willingness to pay (WTP) for a policy alternative or attribute level is indirectly revealed based on the choices they make (e.g., Hanley et al., 2001). The CE method has been found suitable for valuing genetic resources due to its flexibility and ability to value the different traits that breeds or varieties may have. The CE method can also be used to evaluate the means of conservation *in situ* (live animals and plants) and *ex situ* (as seeds, cryopreserved embryos and other genetic material), and both plant genetic resources (PGR) and animal genetic resources (AnGR).

Previous choice experiments have focused on valuing breeds or varieties and their attributes, especially related to their use in agriculture (Birol et al., 2006; Ouma et al., 2007), and applications focusing on consumer or citizen values for AgGR are rare. Valuation studies on biodiversity have found heterogeneity in consumer preferences, and even identified lexicographic preferences toward conservation (Hanley et al., 1995; Sælensminde, 2006). Lexicographic preferences imply that people are unwilling to accept any trade-offs for changes in environmental goods, such as biodiversity, and may arise when an individual believes that the environment should be protected without regard to the costs. In the context of AgGR, preference heterogeneity has mainly been studied among farmers (e.g., Ouma et al., 2007; Omondi et al., 2008; Roessler et al., 2008), and there have been only few empirical studies of heterogeneity of citizen preferences (Zander et al., 2013) or lexicographic preferences.

In this paper, we present the results of a choice experiment conducted to estimate the benefits of genetic resource conservation programmes in Finland. We tested the effect of *in situ* and *ex situ* conservation on citizens' choices between programmes. We also analyzed whether plant varieties and animal breeds are perceived as equally valuable by citizens. As heterogeneity in the preferences for the conservation of AgGR is likely, we tested for the existence of citizen segments that place different values on the conservation of genetic resources.

We expected that AgGR would be rather unfamiliar to some of the respondents of the valuation survey. However, in valuation surveys, respondents are assumed to make “informed” choices when responding to value elicitation questions (e.g., Blomquist and Whitehead, 1998). To obtain informed choices that produce valid estimates of WTP, surveys need to provide a sufficient amount of neutral information on the environmental good while avoiding information overload. Providing more information on the quality (characteristics and services) of an environmental good can increase the stated WTP, have no effect, or in some cases reduce WTP (Blomquist and Whitehead, 1998).

There is a substantial body of literature on the effects of information and respondent effort in contingent valuation studies (e.g., Cameron and Englin, 1997; Blomquist and Whitehead, 1998; Berrens et al., 2004), and some choice experiment studies have also examined the issue, mainly focusing on respondent effort (Hu et al., 2009; Vista et al., 2009). Hu et al. (2009) used data from a choice experiment concerning genetically modified food to simultaneously model voluntary information access and product choices. They found that information was accessed rather infrequently, and that those who held critical views on GM food accessed information more often. There were interlinkages between information access and choices, but they were complex and varied between individuals. Vista et al. (2009) examined the effect of time spent on attribute information, choice questions or completing the survey, finding no significant effects on parameter estimates.

Here, we were particularly interested in examining how the use of information differs between respondent segments. In the survey, respondents had the opportunity to obtain additional information on genetic resources by accessing a hyperlink to a web page. The Internet survey allowed us to measure whether the respondents accessed the additional information and how much time they used to read it. Offering the opportunity for voluntary access to information instead of using different information treatments for split samples has the advantage of not assuming that respondents read all the information that is provided (Hu et al., 2009). Furthermore, we tested the effects of response certainty and self-perceived carefulness in filling the survey as sources of preference heterogeneity.

The rest of the paper is organized as follows. Section Materials and Methods introduces the data and statistical models used in the analysis. Results are presented in section Results, and section Discussion and Conclusions provides discussion and conclusions.

## Materials and methods

### Data collection

The survey data were collected using an Internet survey during the summer of 2011. The sample was drawn from the Internet panel of a private survey company, Taloustutkimus, which comprises 30,000 respondents who have been recruited to the panel using random sampling to represent the population (Taloustutkimus, 2013). After a pilot survey of 138 people, a random sample of 6200 respondents was selected, of which 2426 completed parts of the survey and 1495 completed the survey entirely. These numbers correspond to response rates of 39 and 24%, respectively. Based on the socio-demographic variables, the data represented the population rather well (Table 1).

**Table 1:** **Descriptive statistics (*n* = 1608)**.

	**In the data**	**In the population^a^**
Proportion of females, %	48	51
Mean age, years	52	47
Proportion of people with a higher educational level, %	24	23
Proportion of people living in households with a gross income under €40,000, %	43	53
Proportion of people with children (<18 years) in the family, %	35	40
Proportion of people living in South Finland, %	40	41

a *Statistics Finland 2010, www.stat.fi*.

### Survey design

In the first section, the survey introduced the most common Finnish native animal breeds and plant varieties by explaining what landraces are and giving examples. After asking the respondents about their familiarity with PGR and AnGR, all respondents were offered a short piece of information on the conservation of these breeds and varieties. Next, the respondents were given the opportunity to obtain further information by clicking on two hyperlinks, one for PGR and the other for AnGR. Providing voluntary access to additional information made it possible to identify those respondents who accessed the information, and the time spent on the information page was also recorded (Hu et al., 2009). The additional information provided in our survey included motives for conservation, descriptions of the *in situ* and *ex situ* conservation methods and facts about the sustainable use of genetic resources. After several questions concerning perceptions of genetic resources, the survey proceeded to the choice experiment.

The choice experiment was framed by telling respondents that the conservation of native plant varieties and animal breeds is not yet comprehensive in Finland. The survey presented a programme that would conserve the majority of the varieties and breeds on farms and in gene banks. The operation of gene banks would be extended to missing plants and varieties, and conservation on farms would be enhanced by developing the support provided to farmers for conservation activities. Furthermore, those who are using native varieties in gardens were stated to be supported monetarily and by providing guidance.

The survey explained that the conservation programme would be financed with an increase in income tax between the years 2012 and 2021, and that depending on the extent of the programme, the cost to taxpayers would vary, but all taxpayers would participate in financing the programme. The conservation measures (attributes) of the alternative programmes were illustrated to the respondents using a table.

Table 2 presents the attributes together with their descriptions and levels. The first attribute level is always the level specified in the status quo alternative (current state). The attributes included conservation measures of both plant varieties and animal breeds in gene banks and farms. Instead of having a separate attribute for each native breed, only one attribute for breeds in gene banks and one on farms was included to have the same number of attributes for varieties and breeds, and to ease the cognitive burden of the respondents. The native breeds in gene banks attribute had eight levels and native breeds on farms nine levels, including the status quo attribute level.

**Table 2:** **Attributes of conservation programmes and their levels**.

**Attribute**	**Description**	**Current state**	**Levels (unit)**
Native food plant varieties in gene banks	Native food plants are stored in a gene bank, either as seeds or plant parts.	The gene bank contains seeds from about 300 landrace varieties. Plants that are added vegetatively (e.g., berry and apple varieties) are missing.	300, 400, 500 (number of plants)
Farms growing native food plants	Farmers and hobby gardeners cultivate native food plants on farms or in gardens.	Seven farms grow seeds of native food plants with agri-environmental support. Other activities than growing seeds are not supported.	7, 500, 1000 (number of farms)
Native ornamental plant varieties mapped and in gene banks	Scientists identify and register native ornamental plants. Varieties are preserved in a gene bank, either as seeds or plant parts.	Only a small proportion of the native ornamental plants are known. Storage in the official gene bank is not provided.	small proportion, about half, majority (proportion of plants)
Native breeds in gene banks	Landrace breeds are kept in a gene bank as gametes and embryos.	The gene bank contains Western, Eastern and Northern Finncattle, as well as Finn-, Åland and Kainuu sheep. Native chicken, goat and horse breeds are missing from the gene bank.	3 cattle breeds and 3 sheep breeds (status quo level), + all combinations of goat, horse and chicken breeds
Native breeds on farms	Native breeds are kept on farms in their natural environment. A breed is considered to be endangered if the number of females is less than 1000.	Farms secure goat, horse and chicken breeds, Finnish sheep and Western Finncattle. Eastern and Northern Finncattle, as well as Åland and Kainuu sheep, are endangered.	1 cattle breed, 1 sheep breed, goat, horse and chicken (status quo level), + all combinations of additional 1-2 cattle and sheep breeds
Cost	Cost for taxpayers, €/year during 2012–2021.	No additional costs.	0, 5, 20, 40, 80, 100, 150, 300 (€)

After introducing the attributes, the respondents were presented with six choice tasks. Each choice task included three alternatives: the status quo alternative, described as maintaining the current situation, and two policy alternatives describing an improved level of conservation compared to the current level. Each alternative was described with five conservation attributes, their levels and the cost attribute. The status quo alternative was uniform across choice tasks. An example of a choice task is shown in Table 3.

**Table 3:** **Example of a choice set**.

		**Current state**	**Conservation programme A**	**Conservation programme B**
Native food plant varieties in gene banks	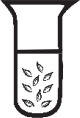	Approximately 300	400	400
Farms growing native food plants	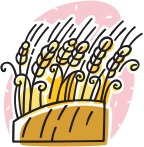	7 farms	2000 farms	1000 farms
Native ornamental plant varieties mapped and in gene banks	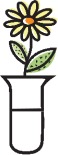	Some	Majority	About half
Native breeds in gene banks	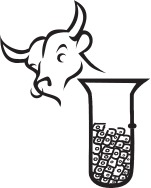	3 cattle breeds, 3 sheep breeds	3 cattle breeds, 3 sheep breeds, chicken, goat, horse	3 cattle breeds, 3 sheep breeds, goat
Native breeds on farms	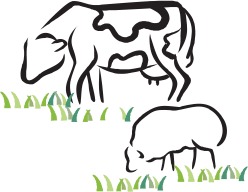	Goat, horse, chicken, 1 cattle breed, 1 sheep breed	Goat, horse, chicken, 3 cattle breeds, 1 sheep breed	Goat, horse, chicken, 2 cattle breeds, 3 sheep breeds
Cost for taxpayers, €/year during 2012–2021	€	€0/year	€80/year	€200/year
I support the alternative		( )	( )	( )

We employed an efficient experimental design to allocate the attribute levels to the choice tasks in the choice experiment survey. Efficient designs aim to generate parameter estimates with standard errors that are as low as possible, and thus produce the maximum information from each choice situation (e.g., Rose and Bliemer, 2009). The generation of efficient designs requires the specification of priors for the parameter estimates. In the pilot survey, we employed zero priors in the design, and used the parameter estimates obtained in the pilot study to construct the final experimental design. In the final study, we employed a Bayesian D-efficient design using Ngene (v. 1.0.2), taking 500 Halton draws for the prior parameter distributions. Bayesian designs take into account the uncertainty related to the parameter priors. Instead of fixed priors, they make use of random priors by specifying a mean and standard deviation for the prior.

In the design phase, animal breeds in gene banks and on farms were treated as separate attributes, but were later combined to the “Native breeds in gene banks” and the “Native breeds on farms” attributes in the choice tasks presented to the respondents. Bayesian priors were employed for the chicken attribute and the number of cattle breeds on the farm attribute, and fixed priors for all other attributes. We generated 180 choice tasks, blocking them into 30 subsets, which resulted in six choice situations presented for each respondent. The final design had a D-error of 0.002.

### Statistical models

The choices between environmental programmes were originally modeled with a conditional logit model (also called a multinomial logit model) (McFadden, 1974). The conditional logit, however, assumes a similar preference structure for all respondents, which implies that they have similar tastes for the attributes of conservation. In this study, we were particularly interested in defining heterogeneous citizen segments, which have a similar preference structure within each segment. One approach that allows this heterogeneity is the latent class model (Boxall and Adamowicz, 2002), which has frequently also been applied in choice experiment models of environmental conservation programmes (e.g., Garrod et al., 2012; Grammatikopoulou et al., 2012). In the latent class model, preferences are assumed to be homogeneous in each segment, but to vary between the segments.

In the modeling, price was treated as a continuous variable and the other attributes were effects-coded, implying that the parameters will sum to zero over the categories of the nominal variable concerned. The status quo attribute levels were thus included in the model, and could obtain either negative or positive coefficients depending on their effect on respondent's utility. Alternative-specific constants (ASC) were included for all alternatives in order to allow systematic choice tendencies not explained with the parameters describing the attributes.

Heterogeneity was statistically included in the latent class model by simultaneously dividing individuals into behavioral groups or latent segments, and estimating a choice model for each of these classes. The estimation was carried out by assuming first one class, then two classes, three classes and so forth. In each step, the explanatory power of the model was assessed to decide on the optimal number of classes. For this purpose, we used the Bayesian information criterion (BIC) and Akaike information criterion (AIC), which are log-likelihood scores with correction factors for the number of observations and the number of parameters. The latent class model also enables the calculation of the WTP for the attributes for each citizen segment.

The relationship between the individual characteristics and the latent classes was examined *a posteriori* of the actual estimation of the latent class model in order to describe the heterogeneous citizen segments. Thus, the segments were formed solely based on the conservation program choices. The membership in the most probable segment was regressed using a logistic regression to characterize each class compared to the rest of the respondents. The explanatory variables for the class memberships included respondents' socioeconomic characteristics, perceived values and responsibilities, use of provided information, response certainty and self-reported perception of the carefulness of completing the survey. The independent variables in the logistic regression models and their descriptive statistics are presented in Table 4.

**Table 4:** **Variables in the logistic regression models**.

**Characteristic**	**Description**	**Mean**	**Standard deviation**	**Min**	**Max**
Female	1 if the respondent is female, if male	0.49	0.50	0	1
Year of birth	Respondents year of birth, continuous	1960	15	1931	1992
High income	1 if household income is over €50,000 per year, 0 otherwise	0.45	0.49	0	1
High education	1 if respondents education level is university education, 0 otherwise	0.24	0.46	0	1
Eastern Finnish	1 if respondents lives in Eastern Finland, 0 otherwise	0.11	0.32	0	1
Childhood in city	1 if respondent spent his/her childhood in a city, 0 otherwise	0.41	0.49	0	1
Certainty	Mean of respondent's certainty in the conservation programme choices, on a scale of 10 completely certain—1 not at all certain.	6.85	2.23	1	10
Agri-environmental attitude	Importance of environmental issues in agriculture, mean of nine measures on scales from 1 to 4	3.26	0.44	1	4
Relative importance of preserving AgGR	The importance of preserving native breeds and varieties relative to other environmental protection measures,	0.94	0.16	0.36	1.66
	1 if both equally important,				
	>1 if preserving native breeds and varieties more important,				
	<1 if other environmental protection measures more important				
Existence value	Factor score based on 8 measures of the importance of existence values, continuous^*^	0.00	1.00	−4.38	2.39
Use values	Factor score based on 8 measures of the importance of use values, continuous^*^	0.00	1.00	−3.78	2.62
Citizen responsibility	Factor score based on 9 measures of stakeholder responsibilities in conservation^*^	0.00	1.00	−3.38	2.30
Consumer responsibility	Factor score based on 9 measures of stakeholder responsibilities in conservation^*^	0.00	1.00	−5.27	2.01
Farmer responsibility	Factor score based on 9 measures of stakeholder responsibilities in conservation^*^	0.00	1.00	−3.12	2.88
Familiarity of products	Familiarity of AgGR products, mean of 10 measures on scales from 1 to 3	2.03	0.42	1	3
Info use (animals) > 0.5 min	1 if respondent used more than 30 s for additional information about breeds, 0 otherwise	0.33	0.47	0	1
Info use (plants) > 0.5 min	1 if respondent used more than 30 s for additional information about varieties, 0 otherwise	0.35	0.48	0	1
Hasty response	1 if respondent evaluated his/her response as hasty, 0 if careful	0.05	0.22	0	1

* *Detailed description of these variables can be found in Tienhaara et al. (2014)*.

## Results

In 24% of the choice sets, the respondents chose the status quo option, i.e., the current state without any additional program to conserve AgGR. The probability of choosing one of the two alternative conservation programs varied between 46% for the lowest cost level of €5 and 28% for the highest cost level of €300.

Table 5 presents the conditional logit model results for the choice of the conservation programme. As expected, an increase in the programme cost negatively affected the probability of choosing it. Turning to consider the genetic resource attributes, the number of food plants in the gene bank was not statistically significant. All other attributes were significant in determining respondents' choices. A higher number of farms growing native plant varieties increased the choice probability. The larger the number of ornamental plants to be mapped and conserved in gene banks, the more probable it was that the respondent would choose the programme. Conserving native breeds of Finnish goats, horses and chickens in the gene bank all increased the support for the programme. The effect was highest for horse, followed by chicken and goat. The guaranteed existence of cattle breeds on farms had a positive and significant effect on choice. As expected, the effect was greater if the number of conserved cattle breeds was three instead of two. This was also the case with sheep breeds, although the conservation of two breeds did not have a positive effect on choice compared with the status quo of one conserved breed.

**Table 5:** **Conditional logit (CL) model results**.

**Variable**	**Coefficient**	**Wald *p*-value**
ASC1 (SQ)	−0.263^***^	0.000
ASC2	0.291^***^	
ASC3	−0.028	
Cost	−0.005^***^	0.000
300 plants in bank (SQ)	0.002	1.000
400 plants in bank	−0.002	
500 plants in bank	0.000	
7 plants on farms (SQ)	−0.199^***^	0.000
500 plants on farms	0.075^***^	
1000 plants on farms	0.124^***^	
Ornamental plants in bank (SQ)	−0.057^**^	0.008
Ornamental plants in bank L2	−0.004	
Ornamental plants in bank L3	0.061^***^	
Goats (SQ)	−0.039^***^	0.005
Goats in bank	0.039^***^	
Horses (SQ)	−0.075^***^	0.000
Horses in bank	0.075^***^	
Chickens (SQ)	−0.047^***^	0.001
Chickens in bank	0.047^***^	
1 cattle breed on farms (SQ)	−0.114^***^	0.000
2 cattle breeds on farms	0.025	
3 cattle breeds on farms	0.089^***^	
1 sheep breed on farms (SQ)	0.020	0.027
2 sheep breeds on farms	−0.052^***^	
3 sheep breeds on farms	0.032	
No. of respondents	1608	
No. of observations	9484	
Correct predictions %	48	
R^2^	0.04	

z-test:

*** 99% significance level;

** 95% significance level.

*SQ, attribute level in the status quo alternative*.

The alternative specific constants (ASC) capture the tendency to choose one of the alternatives which is not explained by the attributes. The negative ASC1 (SQ) coefficient showed the reluctance to choose the status quo alternative regardless of the attribute levels in the policy alternatives. Furthermore, the ASC2 and ASC3 coefficients differed unexpectedly in sign and significance. The positive coefficient for ASC2 and negative for ASC3 indicated that the conservation programme that was presented first received more support. This was surprising, as the programmes were not presented in a specific order in the survey. The model predicted 48% of the choices right, clearly exceeding the probability of correct random choices of 33%, leading to a relatively weak goodness of fit.

The homogeneity of preferences was tested in the estimation of the latent class models. Based on the AIC and BIC, the estimation process showed that a model of five citizen clusters provided the best fit of the data. Table 6 presents the latent class model results with the cluster names, and the logit model for the membership of each cluster is presented in Table 7.

**Table 6:** **Latent class models for conservation programme choice**.

	**Class 1**	**Class 2**	**Class 3**	**Class 4**	**Class 5**	**Overall**	
Pseudo R^2^	0.131	0.288	0.019	0.015	0.472	0.559	
Class size	0.27	0.26	0.17	0.17	0.13		
**Class names**	**Conserva-tionists**	**Bid-sensitive animal conservers**	**Uncertain supporters**	**Status quo preferers**	**Bid sensitives**	**Wald *p*-value**	**Wald (=) *p*-value**
**Attributes**	**Coefficients and significance levels**						
ASC 1 (SQ)	−0.990^***^	−2.937^***^	−0.841^***^	1.668^***^	−0.554^**^	0.000	0.000
ASC 2	0.332^***^	1.499^***^	1.757^***^	−0.414^**^	0.478^***^		
ASC 3	0.658^***^	1.438^***^	−0.916^***^	−1.254^***^	0.076		
Cost	0.000	−0.018^***^	−0.003^*^	−0.001	−0.041^***^	0.000	0.000
300 plants in bank (SQ)	−0.162^***^	0.138^**^	0.018	0.412^**^	−0.322^***^	0.003	0.001
400 plants in bank	0.025	−0.007	0.078	−0.166	0.225^*^		
500 plants in bank	0.137^**^	−0.131^*^	−0.096	−0.245	0.097		
7 plants on farms (SQ)	−0.621^***^	−0.120^*^	−0.261^**^	−0.006	−0.169	0.000	0.000
500 plants on farms	0.125^**^	0.208^***^	0.237^*^	0.003	0.104		
1000 plants on farms	0.496^***^	−0.088	0.024	0.003	0.065		
Ornamental plants in bank (SQ)	−0.462^***^	0.015	0.116	−0.004	−0.332^**^	0.000	0.000
Ornamental plants in bank L2	0.158^***^	0.002	0.023	−0.053	0.16		
Ornamental plants in bank L3	0.304^***^	−0.017	−0.139	0.057	0.172		
Goats (SQ)	−0.063^***^	−0.063^***^	−0.063^***^	−0.063^***^	−0.063^***^	0.001	C.i.
Goats in bank	0.063^***^	0.063^***^	0.063^***^	0.063^***^	0.063^***^		
Horses (SQ)	−0.152^***^	−0.128^***^	−0.075	0.447^***^	−0.256^***^	0.000	0.000
Horses in bank	0.152^***^	0.128^***^	0.075	−0.447^***^	0.256^***^		
Chickens (SQ)	−0.062^***^	−0.062^***^	−0.062^***^	−0.062^***^	−0.062^***^	0.001	C.i.
Chickens in bank	0.062^***^	0.062^***^	0.062^***^	0.062^***^	0.062^***^		
1 cattle breed on farms (SQ)	−0.144^***^	−0.144^***^	−0.144^***^	−0.144^***^	−0.144^***^	0.000	C.i.
2 cattle breeds on farms	0.034	0.034	0.034	0.034	0.034		
3 cattle breeds on farms	0.110^***^	0.110^***^	0.110^***^	0.110^***^	0.110^***^		
1 Sheep breed on farms (SQ)	−0.213^***^	0.046	−0.036	0.581^***^	−0.245^**^	0.000	0.001
2 Sheep breeds on farms	0.056	−0.04	−0.156	−0.282	0.116		
3 Sheep breeds on farms	0.157^***^	−0.007	0.192	−0.300	0.128		
No. of respondents	1608						
No. of observations	9484						
Correct predictions %	85						

z-test:

*** 99% significance level;

** 95% significance level;

* *90% significance level*.

*SQ, attribute level in the status quo alternative*.

*C.i., class independent*.

**Table 7:** **Logistic regression models profiling consumer classes**.

**Class**	**Class 1**	**Class 2**	**Class 3**	**Class 4**	**Class 5**
**Variable**	**Coefficients and significance levels**				
Constant	−2.76^***^	−43.31^***^	48.77^***^	39.90^**^	−29.46^**^
Female	−0.46^***^				
Year of birth		0.02^***^	−0.02^***^	−0.02^**^	0.02^*^
High income			−0.39^**^		
High education				−0.72^***^	
Eastern Finnish			0.40^*^		
Childhood in city				−0.68^**^	
Certainty	0.12^***^		−0.09^**^		−0.08^**^
Agri-environmental attitude	0.37^*^	0.43^**^			
Relative importance of AgGR		−1.482^***^	1.412^**^	−1.82^**^	
Existence values	0.32^***^			−0.50^***^	
Use values	0.38^***^			−0.39^***^	
Citizen responsibility		0.29^***^	0.21^**^	−1.06^***^	−0.43^***^
Consumer responsibility		0.17^**^		−0.31^**^	−0.38^***^
Farmer responsibility	−0.16^**^			0.27^**^	
Familiarity of products					−0.48^**^
Info use (animals) > 0.5 min				−0.39^*^	
Info use (plants) > 0.5 min		0.54^***^	−0.47^***^		
Hasty response			0.70^*^		−1.08^**^
N	1088	1201	1098	1077	1199
Nagelkerke R^2^	0.103	0.083	0.071	0.397	0.104
Chi-squared	81.99	71.44	46.48	252.37	68.25
*p*-Value	0.000	0.000	0.000	0.000	0.000
Correctly classified (cut 0.5)	69.6	71.6	83.9	90.4	86.8

Variables are significant at the

*** *99% level*,

** *95% level*,

* *90% level*.

The latent class model showed that although preferences for some attributes, such as conserving goat and chicken breeds in gene banks and cattle breeds on farms, did not differ significantly between clusters, there was significant heterogeneity in preferences for most of the attributes. The first class, named as “conservationists,” comprised 27% of the respondents. They did not take the personal cost of the conservation programme into account in their decision process, as the coefficient of the cost variable was not significant. Instead, almost all the conservation attributes had significant and positive signs. Contrary to other clusters, most plant-related attributes were significant for conservationists. They also valued the conservation of ornamental plants. Table 5 also shows that this cluster perceived higher use and existence values from genetic resource conservation than respondents in other segments, and also higher than average certainty in their responses to the choice tasks. This class contained more men than women and considered the conservation not to be a responsibility of farmers. For this cluster we also tested the effect gardening as a hobby, but it did not turn out to be significant. Thus, it seems that these respondents did not support the program because of the possible private good aspect of measures to support native varieties in gardens.

The second cluster, covering 26% of the respondents, was named as “bid-sensitive animal conservers.” This group had a higher tendency to choose the improvement programmes compared to the status quo. The coefficient of the bid was significant and the second smallest of all clusters. In this cluster, the emphasis of preferences was on the conservation of animal breeds. The conservation of plant varieties in gene banks was even valued negatively. These respondents perceived more often than average that citizens and consumers should be responsible for the conservation of genetic resources. They also had positive agri-environmental attitudes. Furthermore, the respondents in this cluster used more than the average time to familiarize themselves with the information available in the survey concerning PGR, and they were slightly younger than the average respondent.

A confusing aspect in the third cluster was the large difference between the ASC for the two conservation programmes. This group, comprising 17% of the respondents, had a considerably greater tendency to choose conservation programme A rather than B or the status quo, although this could not be explained by the experimental design and attribute levels. The bid variable followed expectations, but for the other attributes, only plants on farms and the class-independent variables were significant. The logistic regression revealed that members of this cluster were older and had a lower income, and they emphasized the responsibility of citizens in conservation. Geographically, this cluster had more members who lived in Eastern Finland. The respondents in this group were relatively uncertain of their preferences, used the additional information less, and responded, according to their self-evaluation, less carefully than other respondents. As there were random tendencies in their support for a programme (ASC), but they still preferred an increase in several conservation attributes, they were named as “uncertain supporters.”

The fourth class, with 17% of respondents, clearly preferred the status quo option, as the ASC for the programme options were negative. The coefficient of the bid variable was not significant. Among these “status quo preferers,” the choice was consistent with their negative attitudes, as the relative importance of AgGR was low, as well as the perceived existence and use values. Citizens and consumers were less frequently seen as those responsible for conservation; instead, it was perceived as a responsibility of the farmers. This class was characterized by an older age, lower educational level and growing up on a farm.

The fifth class of respondents (13%), named as “bid sensitives,” were the most sensitive to the cost of the programme of all groups. Nevertheless, the ASC revealed that they were interested in conservation, and almost all conservation attributes had significant coefficients. Among these respondents, particularly the *ex situ* conservation of Finnhorse positively affected their choices. In this class, the conservation of genetic resources was not seen as a responsibility of citizens or consumers. The logit model for this group showed that they evaluated themselves as careful respondents but felt somewhat uncertain of their choices. They were younger than average and less familiar with products from traditional breeds and varieties.

WTP for different attributes was calculated based on the conditional logit model and the latent class model for those classes for which the cost coefficient was significant (Table 8). WTPs based on the conditional logit model indicated that plants on farms, cattle breeds and horses were most highly valued. In general, there was substantial variation in WTPs between the classes. In class 3, WTPs were higher due to the low importance of the cost attribute.

**Table 8:** **Annual willingness to pay (in 2009 €) for attributes**.

	**Conditional logit model**	**Latent class model, Class 2**	**Latent class model, Class 3**	**Latent class model, Class 5**
Plants in bank (400)	–	–	–	13
Plants in bank (500)	–	-15	–	–
Plants on farms (500)	60	19	7	–
Plants on farms (1000)	70	–	–	–
Ornamental plants (majority) inventoried and in bank	14	–	–	–
Goats in bank	17	7	105	3
Horses in bank	33	15	–	12
Chickens in bank	20	7	104	3
3 cattle breeds on farms	44	14	211	6
2 sheep breeds on farms	−15	–	–	–

*–, Indicates that the estimate is missing due to the non-significance of the cost coefficient*.

## Discussion and conclusions

The results of a choice experiment concerning agricultural genetic resource policies showed that citizens are interested in the conservation of native breeds and varieties in agriculture. However, there was considerable variation in preferences between citizen segments. Of the five identified groups, two groups covering over half of the respondents had a high interest in the conservation of native breeds and varieties. Respondents in one of the segments clearly preferred the current state of conservation to additional conservation efforts, while one group had a favorable attitude toward conservation if the expenses were on a low level, and respondents in one segment were supportive but wavering in their preferences. The respondent groups were identified based on their preferences for conservation, and they also differed with respect to the use of additional information, their response carefulness and the certainty of the stated WTP.

Similar to previous studies of consumer preferences on biodiversity (e.g., Hanley et al., 1995), we also found lexicographic preferences for conserving AgGR. Those were expressed by the largest group of respondents (27%), as their interest in conservation was high regardless of the costs. Lexicographic choices can occur as a result of simplification if the respondent finds the choice task too difficult to handle or as a result of actual lexicographic preferences (Sælensminde, 2006). In our case, it is difficult to determine whether respondents exhibited lexicographic preferences because they wanted to simplify the choice tasks or because the differences in the attribute levels were large. Respondents in the group which exhibited lexicographic preferences were more certain about their preferences, which supports the phenomenon of actual lexicographic preferences as the reason for their choices. In addition, their positive perceptions concerning the existence and use values of genetic resources support the observation of actual lexicographic preferences.

Due to the preference structures, WTP estimates were only obtained for three respondent groups and some of the attributes. In those groups where the cost variable was significant and meaningful WTP estimates could thus be estimated, the marginal WTPs were considerably lower than the WTPs of the whole sample based on the conditional logit model. This implies that in the whole sample, the results were influenced by the groups that were insensitive to the costs of conservation.

Our results can be compared with those obtained by Zander et al. (2013), who assessed the economic value of conservation programs for two Italian cattle breeds using a choice experiment directed to citizens. Zander et al. (2013) also found preference heterogeneity for most of the attributes of the conservation programs, as well as differences in the sensitivity to the cost attribute. According to their findings, 85% of the respondents supported increased conservation, and the mean WTP was 90€ for conserving each breed. The present results can also be linked to previous results of heterogeneity among farmers using native breeds and varieties. Soini et al. (2012) identified a segment of production-oriented farmers among European cattle breeders that would benefit from increased subsidies for keeping native breeds on farms. If the subsidies were increased to correspond to citizens' WTP, it would help particularly this subsidy-dependent group of farmers.

As the survey was Internet-based, we were able to obtain information on the time used for obtaining additional information about plant and AnGR. These variables, combined with certainty, could partly explain the membership in the latent classes. However, similarly to Hu et al. (2009) and Vista et al. (2009), there were no clear tendencies for the use of information to be associated with a lower or higher WTP. Further research is, however, needed to clarify the associations between preferences, uncertainty and information acquisition in the case of genetic resources.

The results provide implications concerning how to direct the conservation policies for AgGR in Finland. The WTP estimates for the attributes of the conservation programmes indicated that the participants valued particularly *in situ* conservation in the case of PGR, which would also imply the existence of native plant varieties in the landscape. However, a moderate level of this *in situ* conservation would be sufficient, as the highest level increased the WTP only slightly. For the conservation of animal breeds, the results emphasize the importance of *in situ* conservation of cattle breeds. The weak support for the conservation of sheep breeds compared to cattle breeds was understandable, as Finnsheep breeds are less familiar to the public. However, the low, even negative, WTP for the conservation of sheep breeds is in contradiction with the importance of Finnsheep in breeding (e.g., Thomas, 2010). *Ex situ* conservation of those animal breeds that are at present insufficiently protected in gene banks was perceived as important, particularly the conservation of the genetic material of the Finnhorse.

Although the cost-effectiveness of AgGR conservation is case-dependent, some previous studies have recommended *ex situ* conservation in gene banks as a less expensive, less vulnerable and less policy-sensitive method of conservation (Dulloo et al., 2010; Silversides et al., 2012). These cost-effectiveness considerations do not, however, take into account the additional benefits that may be associated with *in situ* conservation, such as the visibility of local breeds and varieties in the landscape or the opportunity to use local breed products. Thus, taking into account citizens' preferences for *in situ* and *ex situ* conservation and using cost-benefit analysis in policy evaluation may shift the priorities of agricultural genetic resource conservation policies.

In this study, the conservation policies were based on equal participation of all citizens, as the policy was financed with taxes. An alternative approach would be to apply market-based incentives, e.g., *payments for environmental services (PES)* for the conservation of genetic resources (McNeely, 2006; Wunder, 2007; Narloch et al., 2011). PES would imply that actors who are major users of the resources are involved in making and adapting rules for conservation markets. For future experiments of PES, our results of the citizen groups that are most interested provide information for identifying the interested parties for the markets of AgGR.

### Conflict of interest statement

The Guest Associate Juha Kantanen declares that, despite being affiliated to the same institution as the authors, the review process was handled objectively and no conflict of interest exists. The authors declare that the research was conducted in the absence of any commercial or financial relationships that could be construed as a potential conflict of interest.
